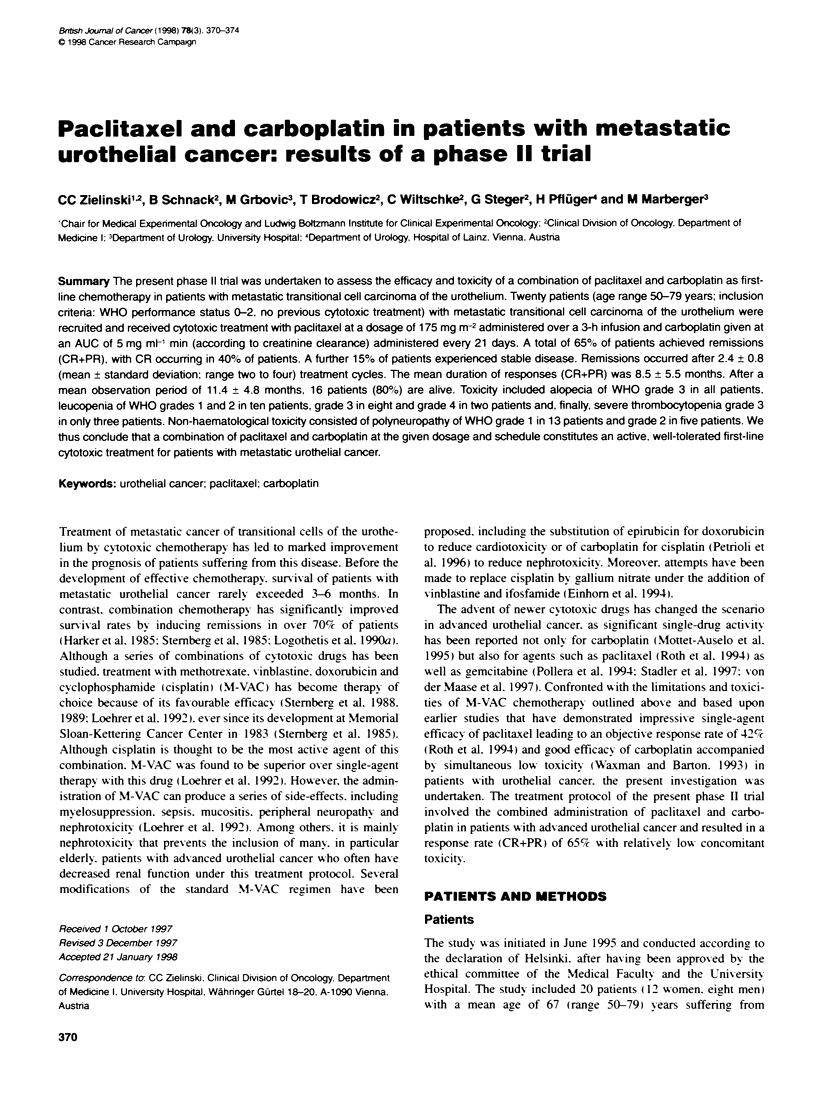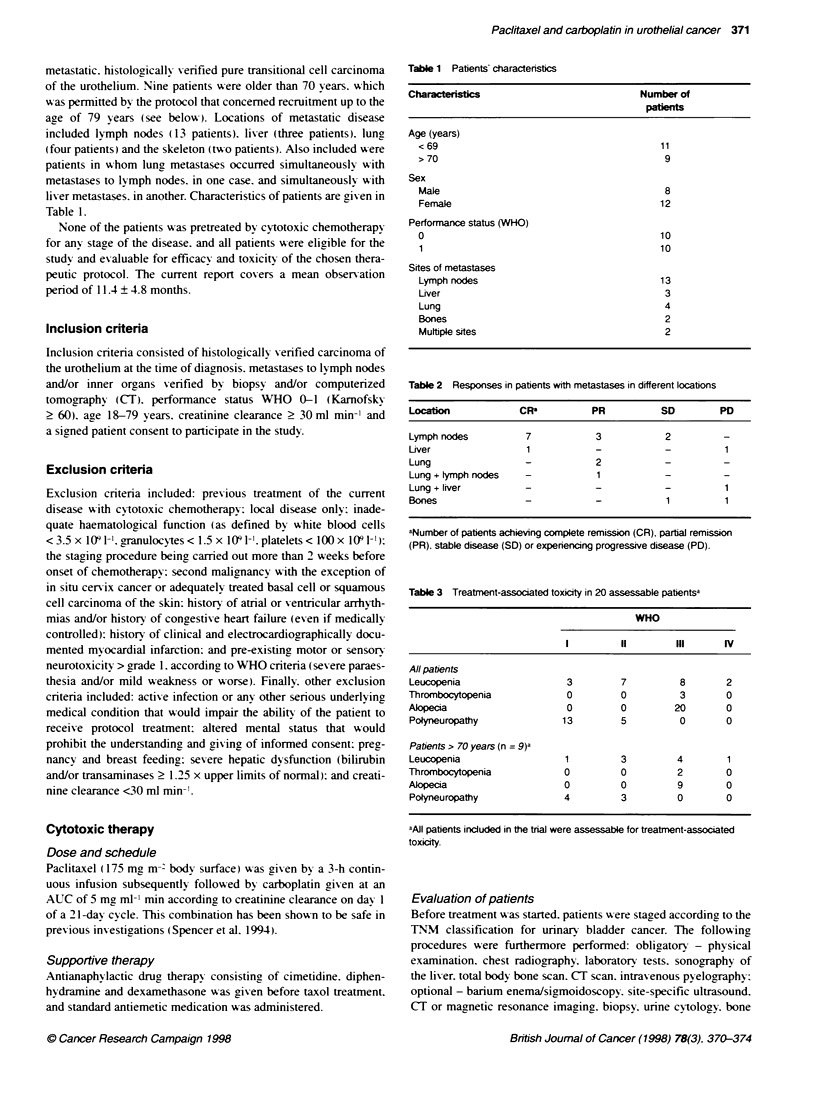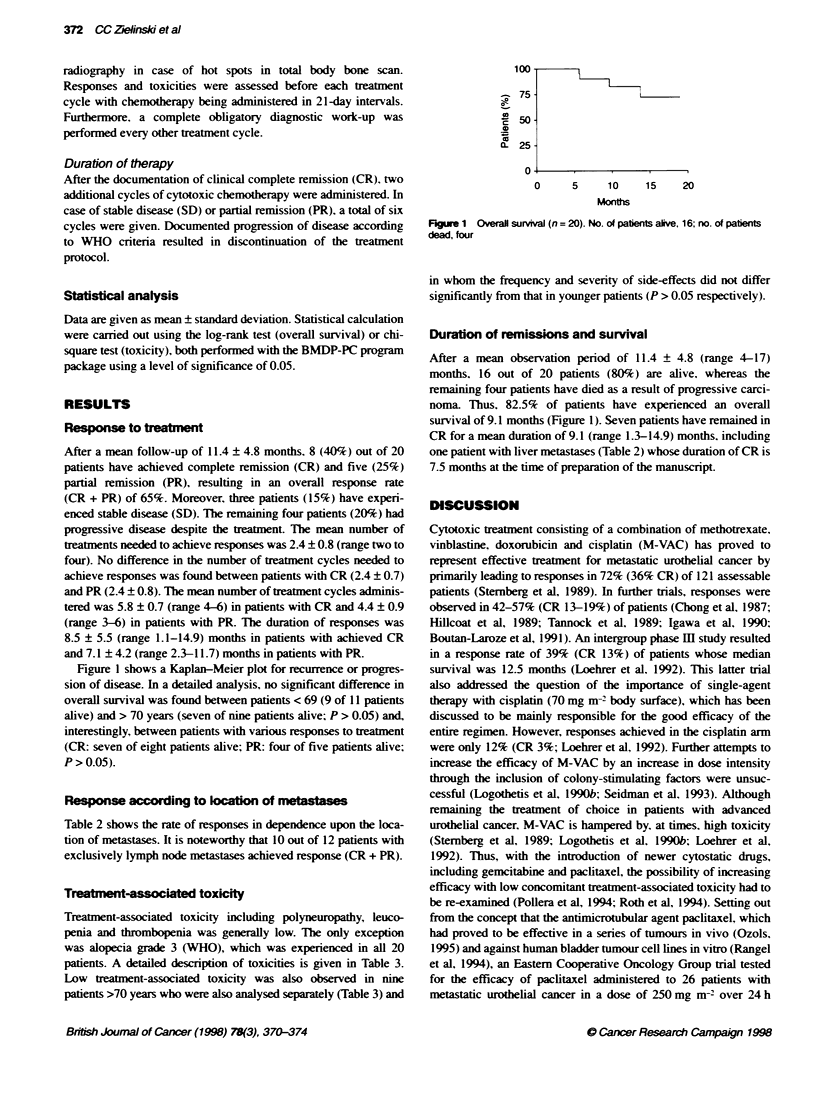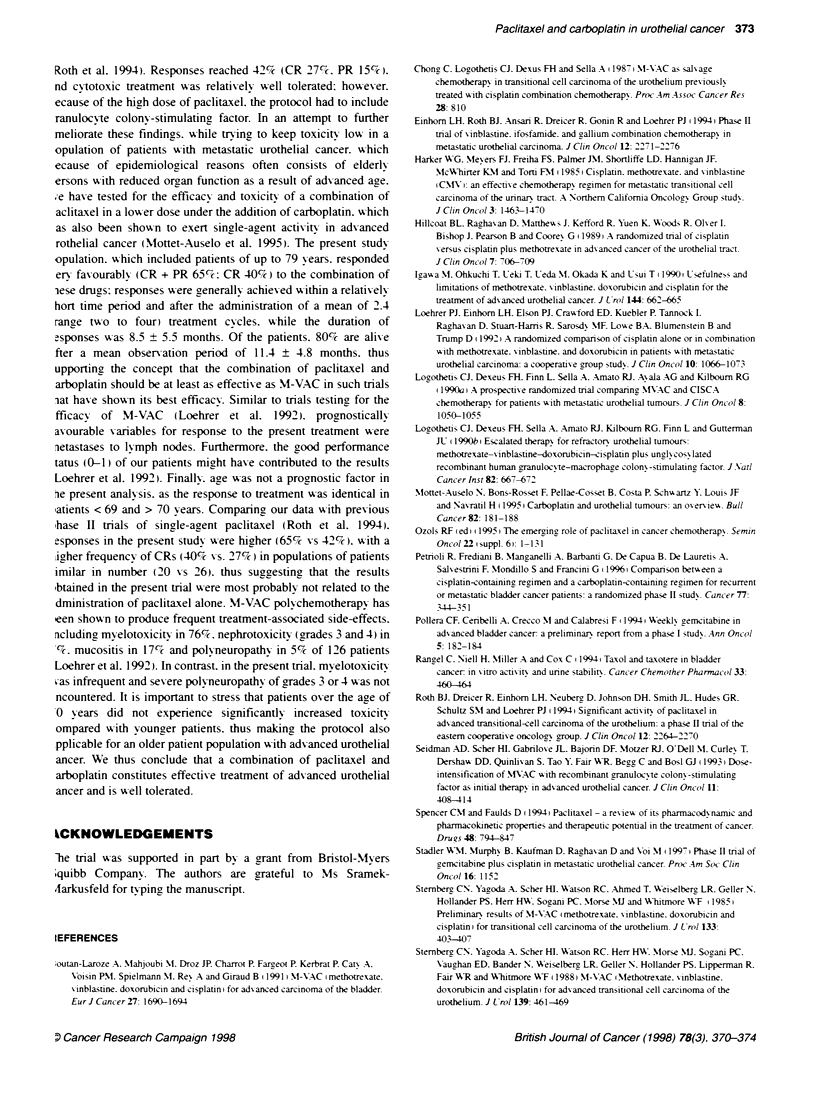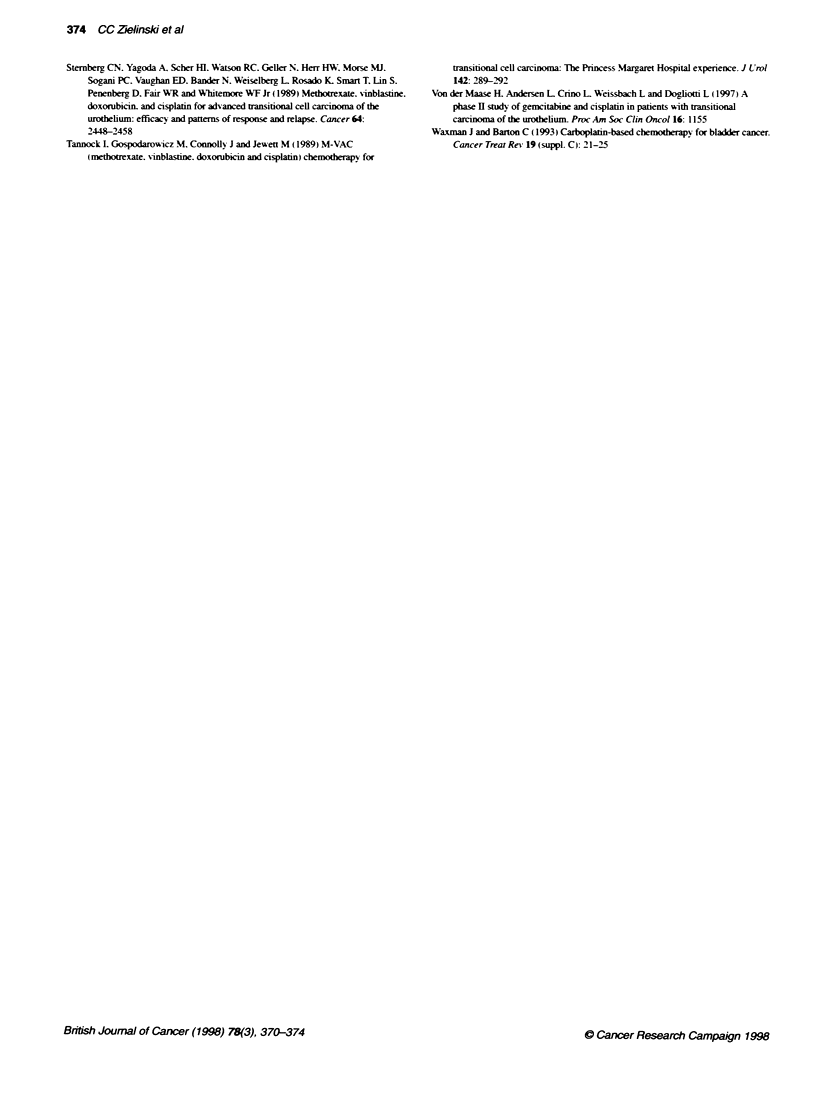# Paclitaxel and carboplatin in patients with metastatic urothelial cancer: results of a phase II trial.

**DOI:** 10.1038/bjc.1998.501

**Published:** 1998-08

**Authors:** C. C. Zielinski, B. Schnack, M. Grbovic, T. Brodowicz, C. Wiltschke, G. Steger, H. Pflüger, M. Marberger

**Affiliations:** Chair for Medical Experimental Oncology and Ludwig Boltzmann Institute for Clinical Experimental Oncology, University Hospital, Vienna, Austria.

## Abstract

The present phase II trial was undertaken to assess the efficacy and toxicity of a combination of paclitaxel and carboplatin as first-line chemotherapy in patients with metastatic transitional cell carcinoma of the urothelium. Twenty patients (age range 50-79 years; inclusion criteria: WHO performance status 0-2, no previous cytotoxic treatment) with metastatic transitional cell carcinoma of the urothelium were recruited and received cytotoxic treatment with paclitaxel at a dosage of 175 mg m(-2) administered over a 3-h infusion and carboplatin given at an AUC of 5 mg ml(-1) min (according to creatinine clearance) administered every 21 days. A total of 65% of patients achieved remissions (CR+PR), with CR occurring in 40% of patients. A further 15% of patients experienced stable disease. Remissions occurred after 2.4 +/- 0.8 (mean +/- standard deviation; range two to four) treatment cycles. The mean duration of responses (CR+PR) was 8.5 +/- 5.5 months. After a mean observation period of 11.4 +/- 4.8 months, 16 patients (80%) are alive. Toxicity included alopecia of WHO grade 3 in all patients, leucopenia of WHO grades 1 and 2 in ten patients, grade 3 in eight and grade 4 in two patients and, finally, severe thrombocytopenia grade 3 in only three patients. Non-haematological toxicity consisted of polyneuropathy of WHO grade 1 in 13 patients and grade 2 in five patients. We thus conclude that a combination of paclitaxel and carboplatin at the given dosage and schedule constitutes an active, well-tolerated first-line cytotoxic treatment for patients with metastatic urothelial cancer.


British Journal of Cancer (1998) 78(3), 370–374
© 1998 Cancer Research Campaign

### Paclitaxel and carboplatin in patients with metastatic urothelial cancer: results of a phase II trial

CC Zielinski[1,2], B Schnack[2], M Grbovic[3], T Brodowicz[2], C Wiltschke[2], G Steger[2], H Pflüger[4] and M Marberger[3]

[1]Chair for Medical Experimental Oncology and Ludwig Boltzmann Institute for Clinical Experimental Oncology; [2]Clinical Division of Oncology, Department of Medicine I; [3]Department of Urology, University Hospital; [4]Department of Urology, Hospital of Lainz, Vienna, Austria

**Summary** The present phase II trial was undertaken to assess the efficacy and toxicity of a combination of paclitaxel and carboplatin as first-line chemotherapy in patients with metastatic transitional cell carcinoma of the urothelium. Twenty patients (age range 50–79 years; inclusion criteria: WHO performance status 0–2, no previous cytotoxic treatment) with metastatic transitional cell carcinoma of the urothelium were recruited and received cytotoxic treatment with paclitaxel at a dosage of 175 mg $m^{-2}$ administered over a 3-h infusion and carboplatin given at an AUC of 5 mg $ml^{-1}$ min (according to creatinine clearance) administered every 21 days. A total of 65% of patients achieved remissions (CR+PR), with CR occurring in 40% of patients. A further 15% of patients experienced stable disease. Remissions occurred after 2.4 ± 0.8 (mean ± standard deviation; range two to four) treatment cycles. The mean duration of responses (CR+PR) was 8.5 ± 5.5 months. After a mean observation period of 11.4 ± 4.8 months, 16 patients (80%) are alive. Toxicity included alopecia of WHO grade 3 in all patients, leucopenia of WHO grades 1 and 2 in ten patients, grade 3 in eight and grade 4 in two patients and, finally, severe thrombocytopenia grade 3 in only three patients. Non-haematological toxicity consisted of polyneuropathy of WHO grade 1 in 13 patients and grade 2 in five patients. We thus conclude that a combination of paclitaxel and carboplatin at the given dosage and schedule constitutes an active, well-tolerated first-line cytotoxic treatment for patients with metastatic urothelial cancer.

**Keywords:** urothelial cancer; paclitaxel; carboplatin

Treatment of metastatic cancer of transitional cells of the urothelium by cytotoxic chemotherapy has led to marked improvement in the prognosis of patients suffering from this disease. Before the development of effective chemotherapy, survival of patients with metastatic urothelial cancer rarely exceeded 3–6 months. In contrast, combination chemotherapy has significantly improved survival rates by inducing remissions in over 70% of patients (Harker et al, 1985; Sternberg et al, 1985; Logothetis et al, 1990*a*). Although a series of combinations of cytotoxic drugs has been studied, treatment with methotrexate, vinblastine, doxorubicin and cyclophosphamide (cisplatin) (M-VAC) has become therapy of choice because of its favourable efficacy (Sternberg et al, 1988, 1989; Loehrer et al, 1992), ever since its development at Memorial Sloan-Kettering Cancer Center in 1983 (Sternberg et al, 1985). Although cisplatin is thought to be the most active agent of this combination, M-VAC was found to be superior over single-agent therapy with this drug (Loehrer et al, 1992). However, the administration of M-VAC can produce a series of side-effects, including myelosuppression, sepsis, mucositis, peripheral neuropathy and nephrotoxicity (Loehrer et al, 1992). Among others, it is mainly nephrotoxicity that prevents the inclusion of many, in particular elderly, patients with advanced urothelial cancer who often have decreased renal function under this treatment protocol. Several modifications of the standard M-VAC regimen have been proposed, including the substitution of epirubicin for doxorubicin to reduce cardiotoxicity or of carboplatin for cisplatin (Petrioli et al, 1996) to reduce nephrotoxicity. Moreover, attempts have been made to replace cisplatin by gallium nitrate under the addition of vinblastine and ifosfamide (Einhorn et al, 1994).

The advent of newer cytotoxic drugs has changed the scenario in advanced urothelial cancer, as significant single-drug activity has been reported not only for carboplatin (Mottet-Auselo et al, 1995) but also for agents such as paclitaxel (Roth et al, 1994) as well as gemcitabine (Pollera et al, 1994; Stadler et al, 1997; von der Maase et al, 1997). Confronted with the limitations and toxicities of M-VAC chemotherapy outlined above and based upon earlier studies that have demonstrated impressive single-agent efficacy of paclitaxel leading to an objective response rate of 42% (Roth et al, 1994) and good efficacy of carboplatin accompanied by simultaneous low toxicity (Waxman and Barton, 1993) in patients with urothelial cancer, the present investigation was undertaken. The treatment protocol of the present phase II trial involved the combined administration of paclitaxel and carboplatin in patients with advanced urothelial cancer and resulted in a response rate (CR+PR) of 65% with relatively low concomitant toxicity.

### PATIENTS AND METHODS

### Patients

The study was initiated in June 1995 and conducted according to the declaration of Helsinki, after having been approved by the ethical committee of the Medical Faculty and the University Hospital. The study included 20 patients (12 women, eight men) with a mean age of 67 (range 50–79) years suffering from

*Received 1 October 1997*
*Revised 3 December 1997*
*Accepted 21 January 1998*

*Correspondence to:* CC Zielinski, Clinical Division of Oncology, Department of Medicine I, University Hospital, Währinger Gürtel 18–20, A-1090 Vienna, Austria metastatic, histologically verified pure transitional cell carcinoma of the urothelium. Nine patients were older than 70 years, which was permitted by the protocol that concerned recruitment up to the age of 79 years (see below). Locations of metastatic disease included lymph nodes (13 patients), liver (three patients), lung (four patients) and the skeleton (two patients). Also included were patients in whom lung metastases occurred simultaneously with metastases to lymph nodes, in one case, and simultaneously with liver metastases, in another. Characteristics of patients are given in Table 1.

None of the patients was pretreated by cytotoxic chemotherapy for any stage of the disease, and all patients were eligible for the study and evaluable for efficacy and toxicity of the chosen therapeutic protocol. The current report covers a mean observation period of 11.4 ± 4.8 months.

### Inclusion criteria

Inclusion criteria consisted of histologically verified carcinoma of the urothelium at the time of diagnosis, metastases to lymph nodes and/or inner organs verified by biopsy and/or computerized tomography (CT), performance status WHO 0–1 (Karnofsky ≥ 60), age 18–79 years, creatinine clearance ≥ 30 ml $min^{-1}$ and a signed patient consent to participate in the study.

### Exclusion criteria

Exclusion criteria included: previous treatment of the current disease with cytotoxic chemotherapy; local disease only; inadequate haematological function (as defined by white blood cells < 3.5 × $10^9$ $l^{-1}$, granulocytes < 1.5 × $10^9$ $l^{-1}$, platelets < 100 × $10^9$ $l^{-1}$); the staging procedure being carried out more than 2 weeks before onset of chemotherapy; second malignancy with the exception of in situ cervix cancer or adequately treated basal cell or squamous cell carcinoma of the skin; history of atrial or ventricular arrhythmias and/or history of congestive heart failure (even if medically controlled); history of clinical and electrocardiographically documented myocardial infarction; and pre-existing motor or sensory neurotoxicity > grade 1, according to WHO criteria (severe paraesthesia and/or mild weakness or worse). Finally, other exclusion criteria included: active infection or any other serious underlying medical condition that would impair the ability of the patient to receive protocol treatment; altered mental status that would prohibit the understanding and giving of informed consent; pregnancy and breast feeding; severe hepatic dysfunction (bilirubin and/or transaminases ≥ 1.25 × upper limits of normal); and creatinine clearance <30 ml $min^{-1}$.

### Cytotoxic therapy

### *Dose and schedule*

Paclitaxel (175 mg $m^{-2}$ body surface) was given by a 3-h continuous infusion subsequently followed by carboplatin given at an AUC of 5 mg $ml^{-1}$ min according to creatinine clearance on day 1 of a 21-day cycle. This combination has been shown to be safe in previous investigations (Spencer et al, 1994).

### *Supportive therapy*

Antianaphylactic drug therapy consisting of cimetidine, diphenhydramine and dexamethasone was given before taxol treatment, and standard antiemetic medication was administered.

**Table 1** Patients' characteristics

| Characteristics | Number of patients |
|---|---|
| Age (years) | |
| < 69 | 11 |
| > 70 | 9 |
| Sex | |
| Male | 8 |
| Female | 12 |
| Performance status (WHO) | |
| 0 | 10 |
| 1 | 10 |
| Sites of metastases | |
| Lymph nodes | 13 |
| Liver | 3 |
| Lung | 4 |
| Bones | 2 |
| Multiple sites | 2 |

**Table 2** Responses in patients with metastases in different locations

| Location | CR[a] | PR | SD | PD |
|---|---|---|---|---|
| Lymph nodes | 7 | 3 | 2 | – |
| Liver | 1 | – | – | 1 |
| Lung | – | 2 | – | – |
| Lung + lymph nodes | – | 1 | – | – |
| Lung + liver | – | – | – | 1 |
| Bones | – | – | 1 | 1 |

[a]Number of patients achieving complete remission (CR), partial remission (PR), stable disease (SD) or experiencing progressive disease (PD).

**Table 3** Treatment-associated toxicity in 20 assessable patients[a]

| | WHO | | | |
|---|---|---|---|---|
| | I | II | III | IV |
| *All patients* | | | | |
| Leucopenia | 3 | 7 | 8 | 2 |
| Thrombocytopenia | 0 | 0 | 3 | 0 |
| Alopecia | 0 | 0 | 20 | 0 |
| Polyneuropathy | 13 | 5 | 0 | 0 |
| *Patients > 70 years* (n = 9)[a] | | | | |
| Leucopenia | 1 | 3 | 4 | 1 |
| Thrombocytopenia | 0 | 0 | 2 | 0 |
| Alopecia | 0 | 0 | 9 | 0 |
| Polyneuropathy | 4 | 3 | 0 | 0 |

[a]All patients included in the trial were assessable for treatment-associated toxicity.

### *Evaluation of patients*

Before treatment was started, patients were staged according to the TNM classification for urinary bladder cancer. The following procedures were furthermore performed: obligatory – physical examination, chest radiography, laboratory tests, sonography of the liver, total body bone scan, CT scan, intravenous pyelography; optional – barium enema/sigmoidoscopy, site-specific ultrasound, CT or magnetic resonance imaging, biopsy, urine cytology, bone

© Cancer Research Campaign 1998

radiography in case of hot spots in total body bone scan. Responses and toxicities were assessed before each treatment cycle with chemotherapy being administered in 21-day intervals. Furthermore, a complete obligatory diagnostic work-up was performed every other treatment cycle.

*Duration of therapy*

After the documentation of clinical complete remission (CR), two additional cycles of cytotoxic chemotherapy were administered. In case of stable disease (SD) or partial remission (PR), a total of six cycles were given. Documented progression of disease according to WHO criteria resulted in discontinuation of the treatment protocol.

### Statistical analysis

Data are given as mean ± standard deviation. Statistical calculation were carried out using the log-rank test (overall survival) or chi-square test (toxicity), both performed with the BMDP-PC program package using a level of significance of 0.05.

### RESULTS

### Response to treatment

After a mean follow-up of 11.4 ± 4.8 months, 8 (40%) out of 20 patients have achieved complete remission (CR) and five (25%) partial remission (PR), resulting in an overall response rate (CR + PR) of 65%. Moreover, three patients (15%) have experienced stable disease (SD). The remaining four patients (20%) had progressive disease despite the treatment. The mean number of treatments needed to achieve responses was 2.4 ± 0.8 (range two to four). No difference in the number of treatment cycles needed to achieve responses was found between patients with CR (2.4 ± 0.7) and PR (2.4 ± 0.8). The mean number of treatment cycles administered was 5.8 ± 0.7 (range 4–6) in patients with CR and 4.4 ± 0.9 (range 3–6) in patients with PR. The duration of responses was 8.5 ± 5.5 (range 1.1–14.9) months in patients with achieved CR and 7.1 ± 4.2 (range 2.3–11.7) months in patients with PR.

Figure 1 shows a Kaplan–Meier plot for recurrence or progression of disease. In a detailed analysis, no significant difference in overall survival was found between patients < 69 (9 of 11 patients alive) and > 70 years (seven of nine patients alive; $P > 0.05$) and, interestingly, between patients with various responses to treatment (CR: seven of eight patients alive; PR: four of five patients alive; $P > 0.05$).

### Response according to location of metastases

Table 2 shows the rate of responses in dependence upon the location of metastases. It is noteworthy that 10 out of 12 patients with exclusively lymph node metastases achieved response (CR + PR).

### Treatment-associated toxicity

Treatment-associated toxicity including polyneuropathy, leucopenia and thrombopenia was generally low. The only exception was alopecia grade 3 (WHO), which was experienced in all 20 patients. A detailed description of toxicities is given in Table 3. Low treatment-associated toxicity was also observed in nine patients >70 years who were also analysed separately (Table 3) and

**Figure 1** Overall survival ($n = 20$). No. of patients alive, 16; no. of patients dead, four in whom the frequency and severity of side-effects did not differ significantly from that in younger patients ($P > 0.05$ respectively).

### Duration of remissions and survival

After a mean observation period of 11.4 ± 4.8 (range 4–17) months, 16 out of 20 patients (80%) are alive, whereas the remaining four patients have died as a result of progressive carcinoma. Thus, 82.5% of patients have experienced an overall survival of 9.1 months (Figure 1). Seven patients have remained in CR for a mean duration of 9.1 (range 1.3–14.9) months, including one patient with liver metastases (Table 2) whose duration of CR is 7.5 months at the time of preparation of the manuscript.

### DISCUSSION

Cytotoxic treatment consisting of a combination of methotrexate, vinblastine, doxorubicin and cisplatin (M-VAC) has proved to represent effective treatment for metastatic urothelial cancer by primarily leading to responses in 72% (36% CR) of 121 assessable patients (Sternberg et al, 1989). In further trials, responses were observed in 42–57% (CR 13–19%) of patients (Chong et al, 1987; Hillcoat et al, 1989; Tannock et al, 1989; Igawa et al, 1990; Boutan-Laroze et al, 1991). An intergroup phase III study resulted in a response rate of 39% (CR 13%) of patients whose median survival was 12.5 months (Loehrer et al, 1992). This latter trial also addressed the question of the importance of single-agent therapy with cisplatin (70 mg $m^{-2}$ body surface), which has been discussed to be mainly responsible for the good efficacy of the entire regimen. However, responses achieved in the cisplatin arm were only 12% (CR 3%; Loehrer et al, 1992). Further attempts to increase the efficacy of M-VAC by an increase in dose intensity through the inclusion of colony-stimulating factors were unsuccessful (Logothetis et al, 1990*b*; Seidman et al, 1993). Although remaining the treatment of choice in patients with advanced urothelial cancer, M-VAC is hampered by, at times, high toxicity (Sternberg et al, 1989; Logothetis et al, 1990*b*; Loehrer et al, 1992). Thus, with the introduction of newer cytostatic drugs, including gemcitabine and paclitaxel, the possibility of increasing efficacy with low concomitant treatment-associated toxicity had to be re-examined (Pollera et al, 1994; Roth et al, 1994). Setting out from the concept that the antimicrotubular agent paclitaxel, which had proved to be effective in a series of tumours in vivo (Ozols, 1995) and against human bladder tumour cell lines in vitro (Rangel et al, 1994), an Eastern Cooperative Oncology Group trial tested for the efficacy of paclitaxel administered to 26 patients with metastatic urothelial cancer in a dose of 250 mg $m^{-2}$ over 24 h

© Cancer Research Campaign 1998

Roth et al. 1994). Responses reached 42% (CR 27%, PR 15%). nd cytotoxic treatment was relatively well tolerated; however, ecause of the high dose of paclitaxel, the protocol had to include ranulocyte colony-stimulating factor. In an attempt to further meliorate these findings, while trying to keep toxicity low in a opulation of patients with metastatic urothelial cancer, which ecause of epidemiological reasons often consists of elderly ersons with reduced organ function as a result of advanced age, ve have tested for the efficacy and toxicity of a combination of aclitaxel in a lower dose under the addition of carboplatin, which as also been shown to exert single-agent activity in advanced rothelial cancer (Mottet-Auselo et al, 1995). The present study opulation, which included patients of up to 79 years, responded ery favourably (CR + PR 65%; CR 40%) to the combination of nese drugs: responses were generally achieved within a relatively hort time period and after the administration of a mean of 2.4 range two to four) treatment cycles, while the duration of esponses was 8.5 ± 5.5 months. Of the patients, 80% are alive fter a mean observation period of 11.4 ± 4.8 months, thus upporting the concept that the combination of paclitaxel and arboplatin should be at least as effective as M-VAC in such trials hat have shown its best efficacy. Similar to trials testing for the fficacy of M-VAC (Loehrer et al, 1992), prognostically avourable variables for response to the present treatment were netastases to lymph nodes. Furthermore, the good performance tatus (0–1) of our patients might have contributed to the results Loehrer et al, 1992). Finally, age was not a prognostic factor in he present analysis, as the response to treatment was identical in atients < 69 and > 70 years. Comparing our data with previous hase II trials of single-agent paclitaxel (Roth et al, 1994), esponses in the present study were higher (65% vs 42%), with a igher frequency of CRs (40% vs. 27%) in populations of patients imilar in number (20 vs 26), thus suggesting that the results btained in the present trial were most probably not related to the dministration of paclitaxel alone. M-VAC polychemotherapy has een shown to produce frequent treatment-associated side-effects, ncluding myelotoxicity in 76%, nephrotoxicity (grades 3 and 4) in %, mucositis in 17% and polyneuropathy in 5% of 126 patients Loehrer et al, 1992). In contrast, in the present trial, myelotoxicity vas infrequent and severe polyneuropathy of grades 3 or 4 was not ncountered. It is important to stress that patients over the age of 0 years did not experience significantly increased toxicity ompared with younger patients, thus making the protocol also pplicable for an older patient population with advanced urothelial ancer. We thus conclude that a combination of paclitaxel and arboplatin constitutes effective treatment of advanced urothelial ancer and is well tolerated.

### ACKNOWLEDGEMENTS

he trial was supported in part by a grant from Bristol-Myers quibb Company. The authors are grateful to Ms Sramek-Iarkusfeld for typing the manuscript.

### EFERENCES

outan-Laroze A, Mahjoubi M, Droz JP, Charrot P, Fargeot P, Kerbrat P, Caty A, Voisin PM, Spielmann M, Rey A and Giraud B (1991) M-VAC (methotrexate, vinblastine, doxorubicin and cisplatin) for advanced carcinoma of the bladder. *Eur J Cancer* **27**: 1690–1694

Chong C, Logothetis CJ, Dexus FH and Sella A (1987) M-VAC as salvage chemotherapy in transitional cell carcinoma of the urothelium previously treated with cisplatin combination chemotherapy. *Proc Am Assoc Cancer Res* **28**: 810

Einhorn LH, Roth BJ, Ansari R, Dreicer R, Gonin R and Loehrer PJ (1994) Phase II trial of vinblastine, ifosfamide, and gallium combination chemotherapy in metastatic urothelial carcinoma. *J Clin Oncol* **12**: 2271–2276

Harker WG, Meyers FJ, Freiha FS, Palmer JM, Shortliffe LD, Hannigan JF, McWhirter KM and Torti FM (1985) Cisplatin, methotrexate, and vinblastine (CMV): an effective chemotherapy regimen for metastatic transitional cell carcinoma of the urinary tract. A Northern California Oncology Group study. *J Clin Oncol* **3**: 1463–1470

Hillcoat BL, Raghavan D, Matthews J, Kefford R, Yuen K, Woods R, Olver I, Bishop J, Pearson B and Coorey G (1989) A randomized trial of cisplatin versus cisplatin plus methotrexate in advanced cancer of the urothelial tract. *J Clin Oncol* **7**: 706–709

Igawa M, Ohkuchi T, Ueki T, Ueda M, Okada K and Usui T (1990) Usefulness and limitations of methotrexate, vinblastine, doxorubicin and cisplatin for the treatment of advanced urothelial cancer. *J Urol* **144**: 662–665

Loehrer PJ, Einhorn LH, Elson PJ, Crawford ED, Kuebler P, Tannock I, Raghavan D, Stuart-Harris R, Sarosdy MF, Lowe BA, Blumenstein B and Trump D (1992) A randomized comparison of cisplatin alone or in combination with methotrexate, vinblastine, and doxorubicin in patients with metastatic urothelial carcinoma: a cooperative group study. *J Clin Oncol* **10**: 1066–1073

Logothetis CJ, Dexeus FH, Finn L, Sella A, Amato RJ, Ayala AG and Kilbourn RG (1990*a*) A prospective randomized trial comparing MVAC and CISCA chemotherapy for patients with metastatic urothelial tumours. *J Clin Oncol* **8**: 1050–1055

Logothetis CJ, Dexeus FH, Sella A, Amato RJ, Kilbourn RG, Finn L and Gutterman JU (1990*b*) Escalated therapy for refractory urothelial tumours: methotrexate–vinblastine–doxorubicin–cisplatin plus unglycosylated recombinant human granulocyte–macrophage colony-stimulating factor. *J Natl Cancer Inst* **82**: 667–672

Mottet-Auselo N, Bons-Rosset F, Pellae-Cosset B, Costa P, Schwartz Y, Louis JF and Navratil H (1995) Carboplatin and urothelial tumours: an overview. *Bull Cancer* **82**: 181–188

Ozols RF (ed) (1995) The emerging role of paclitaxel in cancer chemotherapy. *Semin Oncol* **22** (suppl. 6): 1–131

Petrioli R, Frediani B, Manganelli A, Barbanti G, De Capua B, De Lauretis A, Salvestrini F, Mondillo S and Francini G (1996) Comparison between a cisplatin-containing regimen and a carboplatin-containing regimen for recurrent or metastatic bladder cancer patients: a randomized phase II study. *Cancer* **77**: 344–351

Pollera CF, Ceribelli A, Crecco M and Calabresi F (1994) Weekly gemcitabine in advanced bladder cancer: a preliminary report from a phase I study. *Ann Oncol* **5**: 182–184

Rangel C, Niell H, Miller A and Cox C (1994) Taxol and taxotere in bladder cancer: in vitro activity and urine stability. *Cancer Chemother Pharmacol* **33**: 460–464

Roth BJ, Dreicer R, Einhorn LH, Neuberg D, Johnson DH, Smith JL, Hudes GR, Schultz SM and Loehrer PJ (1994) Significant activity of paclitaxel in advanced transitional-cell carcinoma of the urothelium: a phase II trial of the eastern cooperative oncology group. *J Clin Oncol* **12**: 2264–2270

Seidman AD, Scher HI, Gabrilove JL, Bajorin DF, Motzer RJ, O'Dell M, Curley T, Dershaw DD, Quinlivan S, Tao Y, Fair WR, Begg C and Bosl GJ (1993) Dose-intensification of MVAC with recombinant granulocyte colony-stimulating factor as initial therapy in advanced urothelial cancer. *J Clin Oncol* **11**: 408–414

Spencer CM and Faulds D (1994) Paclitaxel – a review of its pharmacodynamic and pharmacokinetic properties and therapeutic potential in the treatment of cancer. *Drugs* **48**: 794–847

Stadler WM, Murphy B, Kaufman D, Raghavan D and Voi M (1997) Phase II trial of gemcitabine plus cisplatin in metastatic urothelial cancer. *Proc Am Soc Clin Oncol* **16**: 1152

Sternberg CN, Yagoda A, Scher HI, Watson RC, Ahmed T, Weiselberg LR, Geller N, Hollander PS, Herr HW, Sogani PC, Morse MJ and Whitmore WF (1985) Preliminary results of M-VAC (methotrexate, vinblastine, doxorubicin and cisplatin) for transitional cell carcinoma of the urothelium. *J Urol* **133**: 403–407

Sternberg CN, Yagoda A, Scher HI, Watson RC, Herr HW, Morse MJ, Sogani PC, Vaughan ED, Bander N, Weiselberg LR, Geller N, Hollander PS, Lipperman R, Fair WR and Whitmore WF (1988) M-VAC (Methotrexate, vinblastine, doxorubicin and cisplatin) for advanced transitional cell carcinoma of the urothelium. *J Urol* **139**: 461–469

© Cancer Research Campaign 1998

Sternberg CN, Yagoda A, Scher HI, Watson RC, Geller N, Herr HW, Morse MJ, Sogani PC, Vaughan ED, Bander N, Weiselberg L, Rosado K, Smart T, Lin S, Penenberg D, Fair WR and Whitemore WF Jr (1989) Methotrexate, vinblastine, doxorubicin, and cisplatin for advanced transitional cell carcinoma of the urothelium: efficacy and patterns of response and relapse. *Cancer* **64**: 2448–2458

Tannock I, Gospodarowicz M, Connolly J and Jewett M (1989) M-VAC (methotrexate, vinblastine, doxorubicin and cisplatin) chemotherapy for transitional cell carcinoma: The Princess Margaret Hospital experience. *J Urol* **142**: 289–292

Von der Maase H, Andersen L, Crino L, Weissbach L and Dogliotti L (1997) A phase II study of gemcitabine and cisplatin in patients with transitional carcinoma of the urothelium. *Proc Am Soc Clin Oncol* **16**: 1155

Waxman J and Barton C (1993) Carboplatin-based chemotherapy for bladder cancer. *Cancer Treat Rev* **19** (suppl. C): 21–25

© Cancer Research Campaign 1998